# Supplementary figures and images for: Regulation of host immunity by a novel Legionella pneumophila E3 ubiquitin ligase
Source: PLoS Pathog. 2025 Sep 15;21(9):e1013522. doi: 10.1371/journal.ppat.1013522 (PMC12445743; doi:10.1371/journal.ppat.1013522)

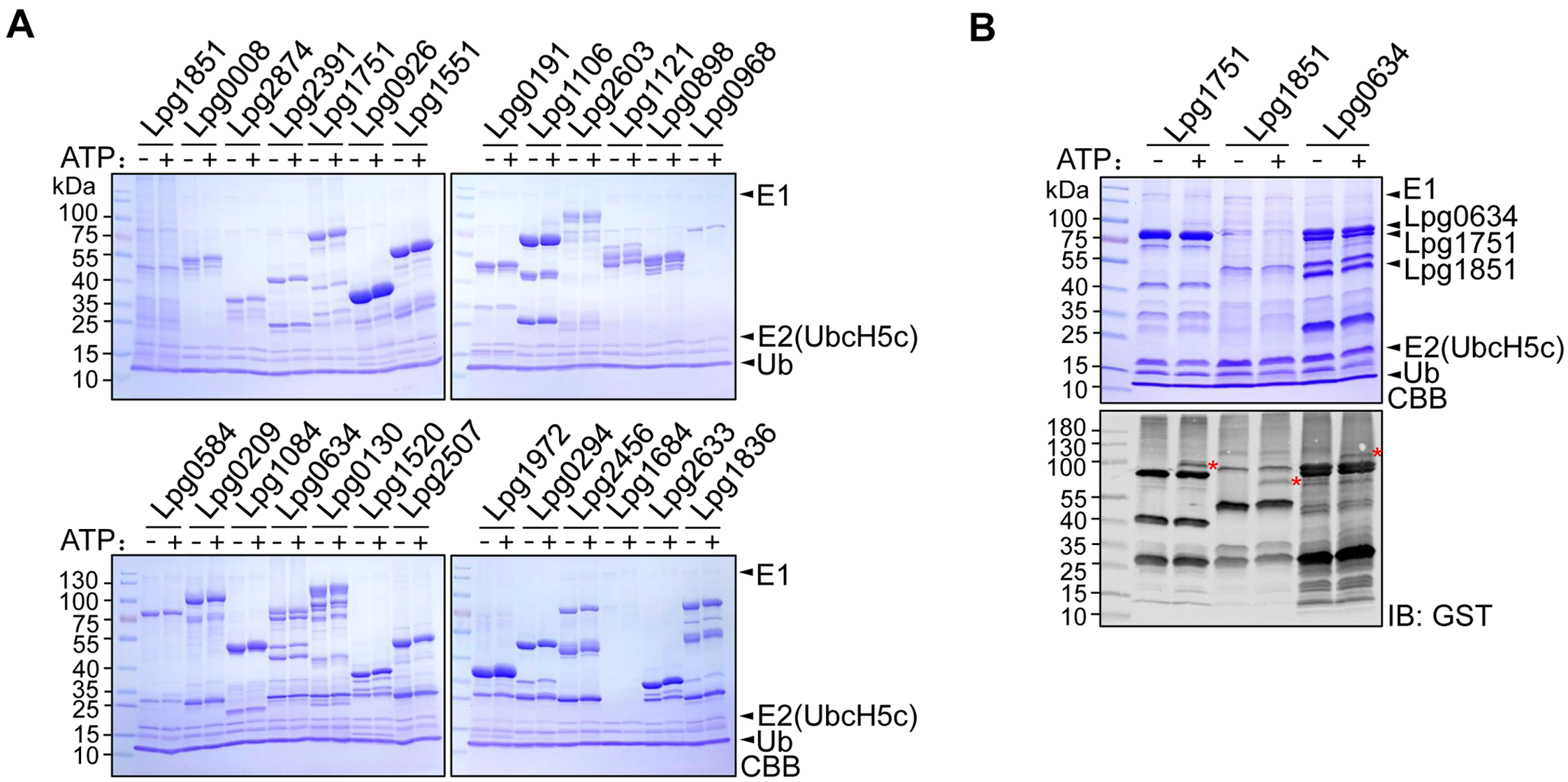

Supplement: S1 Fig — A. Assessment of E3 ligase activity of the 26 candidates using self-modification as a readout. E1, UbcH5c, ubiquitin and GST-tagged effectors were incubated with or without ATP at 37°C for 16 h. Proteins were separated by SDS-PAGE and visualized by CBB staining. B. Lpg1751, Lpg1851 and Lpg0634 displayed weak but detectable self-ubiquitination activity in biochemical assays. After SDS-PAGE, proteins were visualized by CBB staining or immunoblotting with antibodies specific to GST. Self-ubiquitination was determined by the production of protein species with MW higher than their native forms. (TIF) [file ppat.1013522.s001.tif]

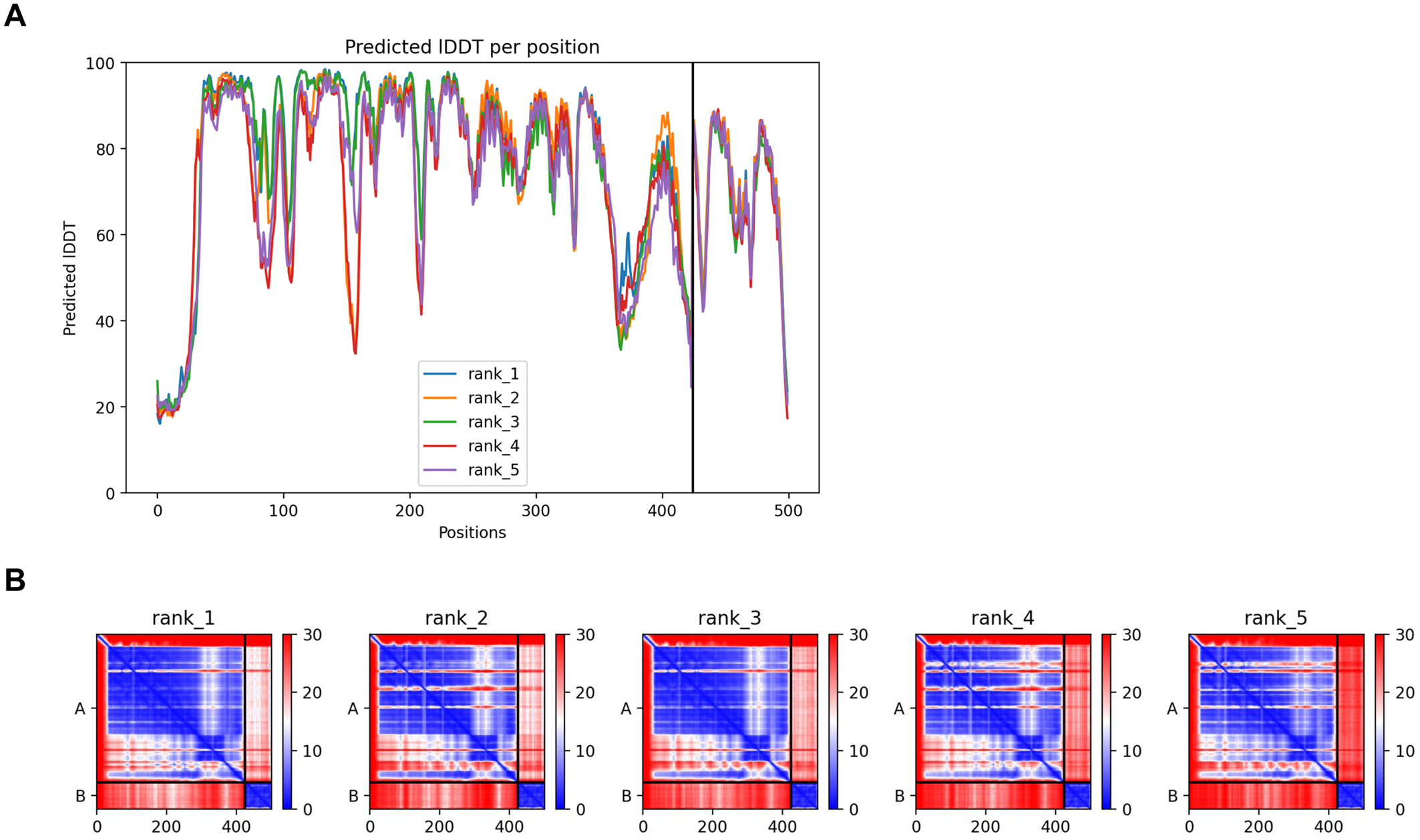

Supplement: S2 Fig — A. The per-residue local Distance Difference Test (IDDT) scores for the predicted Lug14-Ub complex models. B. The PAE metrics for the predicted Lug14-Ub complex models show the predicted relative position error for each residue sequence, with low-confidence values in red and high-confidence values in blue. (TIF) [file ppat.1013522.s002.tif]

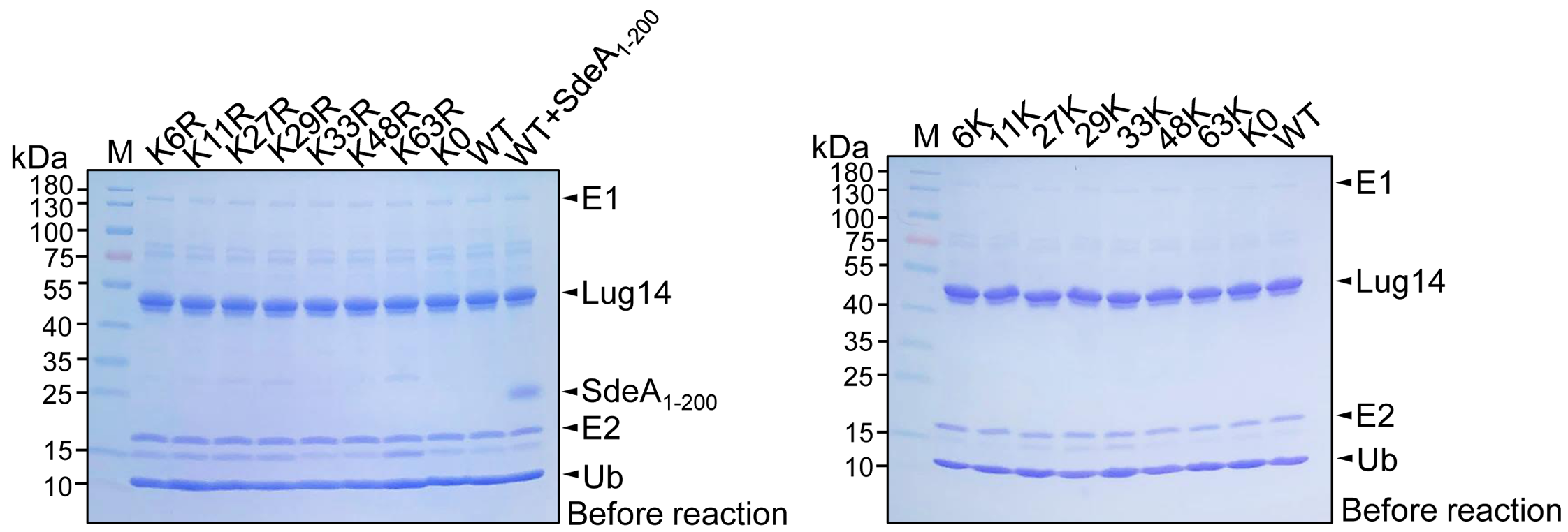

Supplement: S3 Fig — Biochemical ubiquitination assays using E1, UbcH5c, Lug14 and a series of ubiquitin mutants that either carried only a single mutation in one of the lysine residues (left) or retaining only one lysine residue (right). K0 represents the ubiquitin mutant lacking all seven lysine residues. All reactants were added into test tubes in ice, half of the sample in each reaction were taken to determine the amount of Ub detected by Coomassie brilliant blue staining. (TIF) [file ppat.1013522.s003.tif]

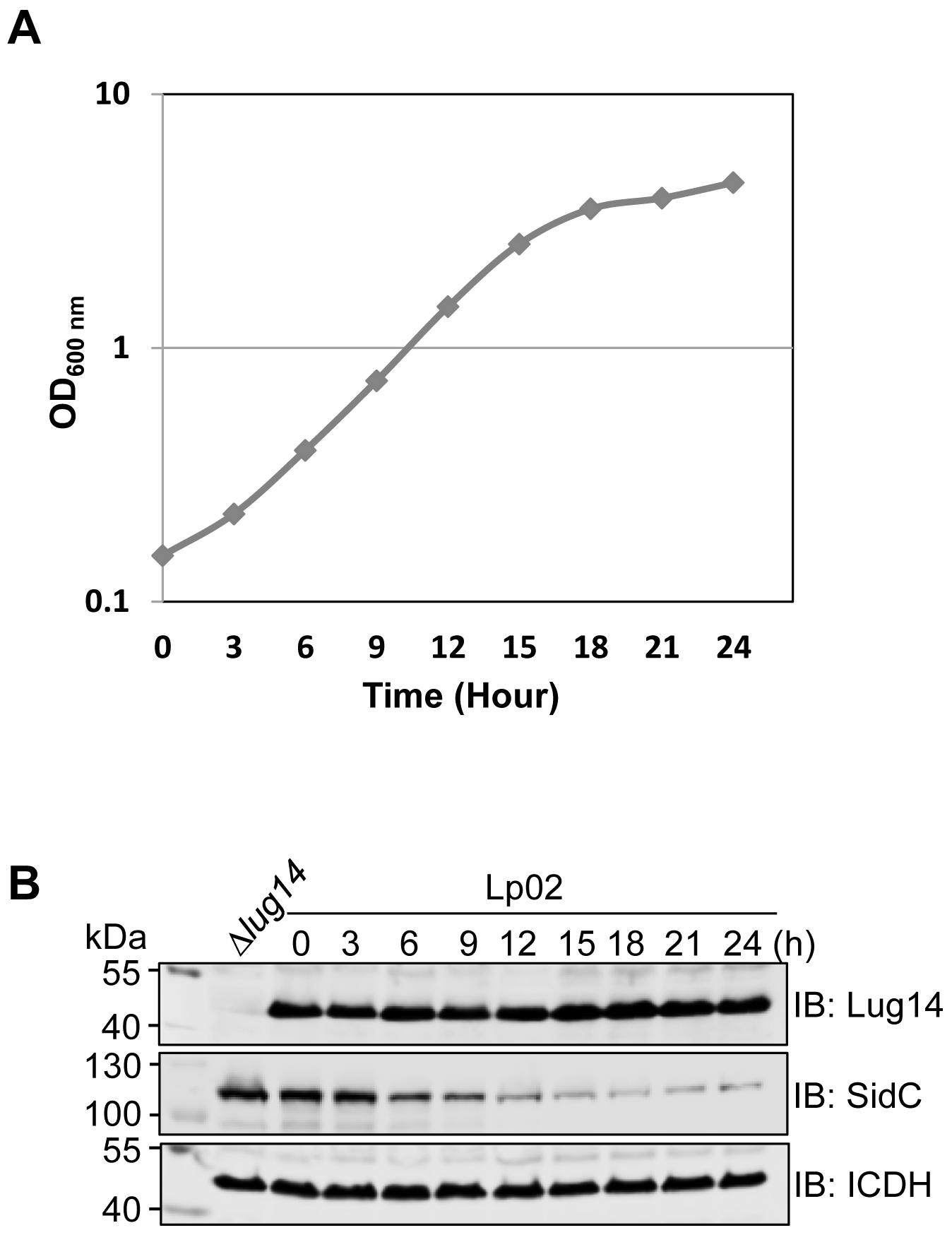

Supplement: S4 Fig — Saturated cultures of L. pneumophila strain Lp02 were diluted in AYE medium to OD600 of 0.1 and the subcultures were grown at 37°C for 24 h. Bacterial growth was determined by measuring OD600 (A) and samples withdrawn at intervals of 3 h were probed for Lug14 by immunoblotting (B). Lysates of the Lp02∆lug14 mutant grown to post exponential phase were included as a control. ICDH was probed as a loading control. (TIF) [file ppat.1013522.s004.tif]

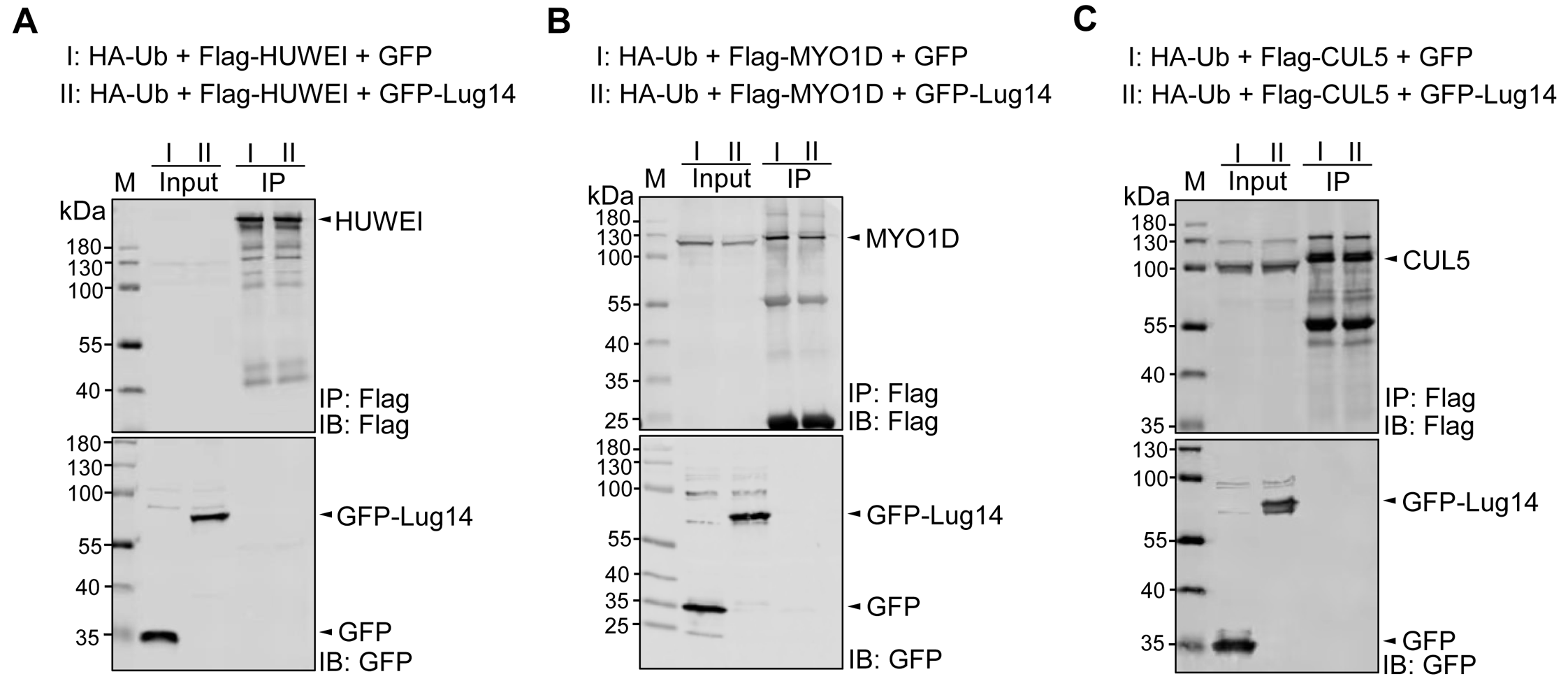

Supplement: S5 Fig — HEK293T cells were co-transfected with plasmids encoding HA-ubiquitin, GFP-Lug14, Flag-tagged HUWEI, MYO1D or CUL5. Samples receiving GFP were established as controls. After SDS-PAGE, samples were probed by immunoblotting with antibodies specific to Flag and GFP, respectively. (TIF) [file ppat.1013522.s005.tif]

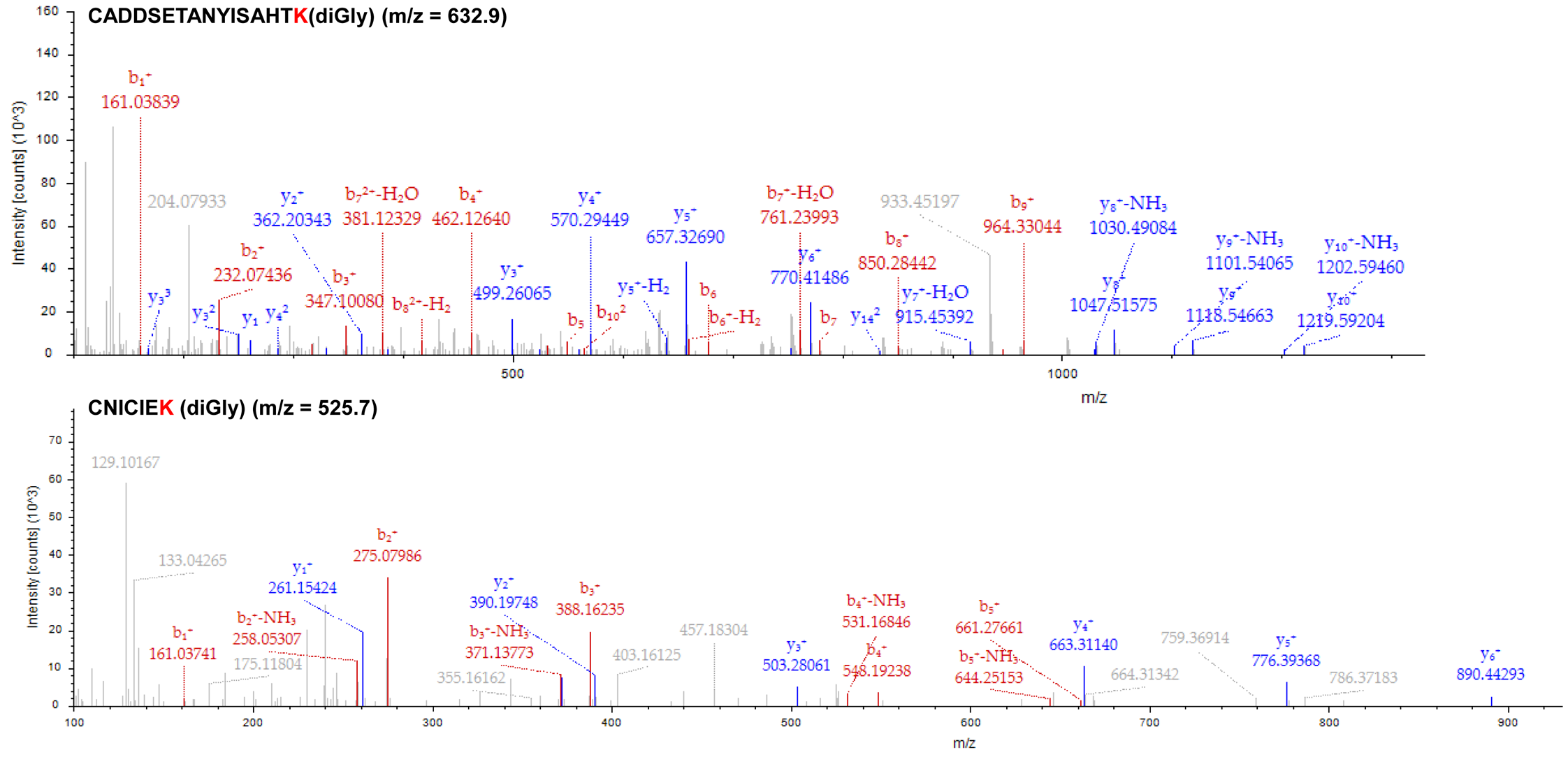

Supplement: S6 Fig — Protein bands representing ubiquitinated ARIH2 were excised and digested with trypsin. The peptides characteristic of ubiquitination bearing the diGly remnant were detected and the modified lysine residues were mapped by LC/MS analysis. The spectra of fragments harboring K295 and K306 were shown. (TIF) [file ppat.1013522.s006.tif]

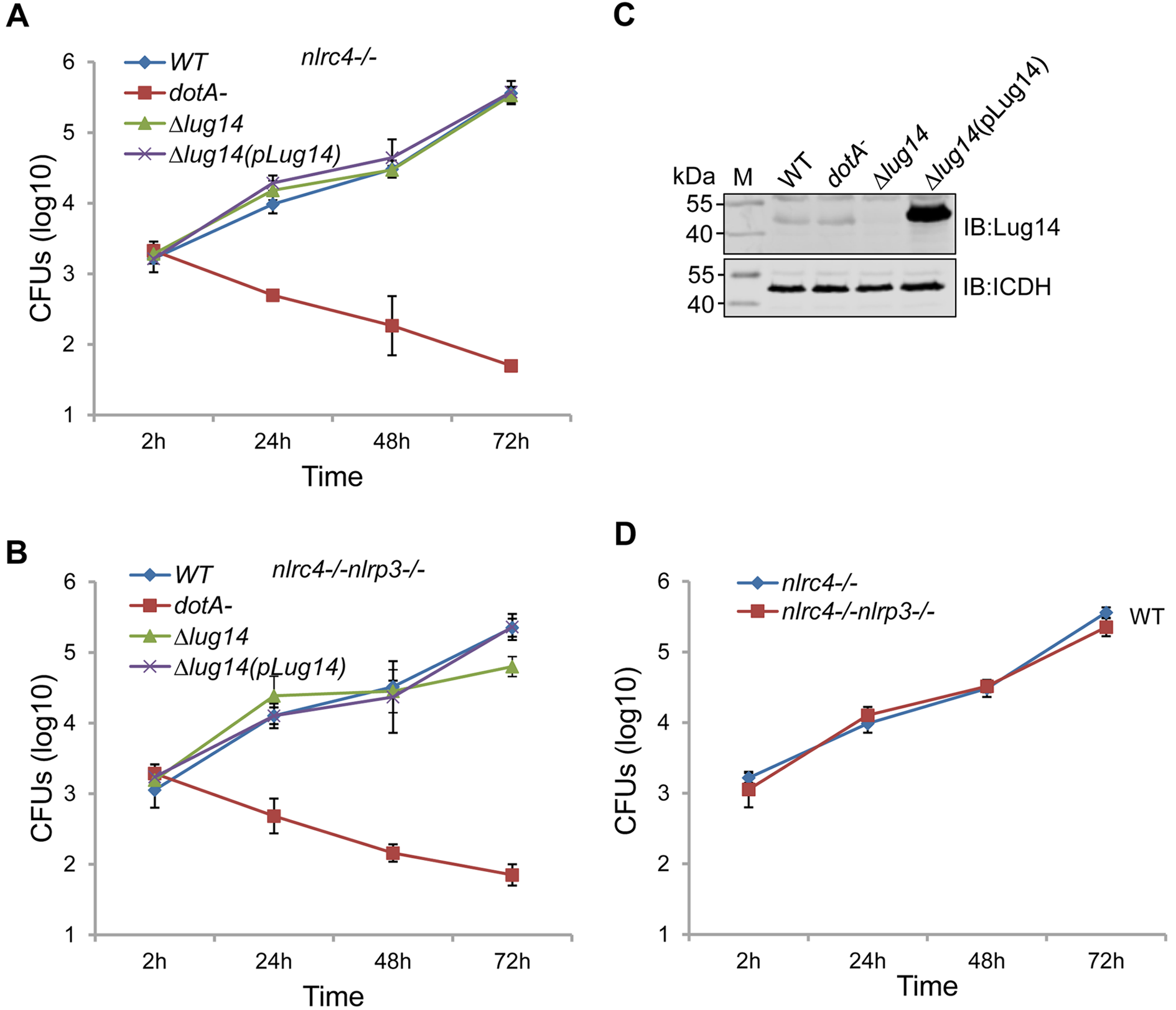

Supplement: S7 Fig — BMDMs from NLRC4−/− (A) or NLRC4−/−/NLRP3−/− (B) mice were infected with the indicated L. pneumophila strains at an MOI of 0.05. Colony-forming-unit of the indicated times points were determined by spotting appropriately diluted lysates of infected cells onto bacteriological medium. Data shown are mean ± s.e. from three samples of each strain. Similar results were obtained in three independent experiments. The expression of lug14 in L. pneumophila strains is used for intracellular growth experiments. Equal amounts of bacterial cells were lysed and proteins resolved by SDS-PAGE were detected by immunoblotting with antibodies specific for Lug14, the metabolic enzyme isocitrate dehydrogenase (ICDH) was probed as a loading control (C). The wild-type strain grew indistinguishably in both hosts (D). (TIF) [file ppat.1013522.s007.tif]
