# Supplementary material for: Regulation of host immunity by a novel Legionella pneumophila E3 ubiquitin ligase
Source: PLoS Pathog. 2025 Sep 15;21(9):e1013522. doi: 10.1371/journal.ppat.1013522 (PMC12445743; doi:10.1371/journal.ppat.1013522)
Supplement: S1 Table — (DOCX) [file ppat.1013522.s008.docx]

**S1 Table *L. pneumophila* proteins that potentially interact with ubiquitin identified by the proximity labelling screen**

| Rank | Accession | Description | Total spectral counts | Unique spectral counts | | |
| --- | --- | --- | --- | --- | --- | --- |
|  |  |  |  | Control | Ub | |
| 1 | Q5ZSK6 | Lpg2511 (SidC) | 2292 | 1179 | 1113 |  |
| 2 | Q5ZSK8 | Lpg2509 (SidD) | 1858 | 836 | 1022 |  |
| 3 | Q5ZUE7 | Lpg1851 | 1510 | 589 | 921 |  |
| 4 | Q5ZXU7 | Lpg0634 | 1441 | 596 | 845 |  |
| 5 | Q5ZSN5 | Lpg2482 (SdbC) | 1311 | 304 | 1007 |  |
| 6 | Q5ZRK7 | Lpg2874 | 1022 | 320 | 702 |  |
| 7 | Q5ZS86 | Lpg2633 | 787 | 348 | 439 |  |
| 8 | Q5ZTK4 | Lpg2157 (SdeA) | 727 | 240 | 487 |  |
| 9 | Q5ZXZ6 | Lpg0584 | 662 | 407 | 255 |  |
| 10 | Q5ZTK6 | Lpg2155 (SidJ) | 635 | 182 | 453 |  |
| 11 | Q5ZTK7 | Lpg2154 (DupA) | 553 | 220 | 333 |  |
| 12 | Q5ZSR1 | Lpg2456 | 489 | 300 | 189 |  |
| 13 | Q5ZSI8 | Lpg2529 (Lem27) | 483 | 165 | 318 |  |
| 14 | Q5ZZK2 | Lpg0008 | 452 | 110 | 342 |  |
| 15 | Q5ZV89 | Lpg1551 | 438 | 236 | 202 |  |
| 16 | Q5ZUP2 | Lpg1751 | 347 | 79 | 268 |  |
| 17 | Q5ZSE2 | Lpg2577 (MavM) | 291 | 2 | 289 |  |
| 18 | Q5ZYR7 | Lpg0294 | 265 | 15 | 250 |  |
| 19 | Q5ZWG4 | Lpg1121 | 253 | 28 | 225 |  |
| 20 | Q5ZX07 | Lpg0926 | 237 | 6 | 231 |  |
| 21 | Q5ZWK1 | Lpg1084 | 187 | 92 | 95 |  |
| 22 | Q5ZZ02 | Lpg0209 | 171 | 34 | 137 |  |
| 23 | Q5ZUG2 | Lpg1836 | 170 | 57 | 113 |  |
| 24 | Q5ZZ20 | Lpg0191 | 168 | 3 | 165 |  |
| 25 | Q5ZSB6 | Lpg2603 | 162 | 99 | 63 |  |
| 26 | Q5ZZ81 | Lpg0130 | 156 | 74 | 82 |  |
| 27 | Q5ZTL4 | Lpg2147 (MavC) | 153 | 106 | 47 |  |
| 28 | Q5ZTK8 | Lpg2153 (SdeC) | 149 | 23 | 126 |  |
| 29 | Q5ZUV8 | Lpg1684 | 146 | 71 | 75 |  |
| 30 | Q5ZWH9 | Lpg1106 | 141 | 32 | 109 |  |
| 31 | Q5ZWW6 | Lpg0968 | 134 | 86 | 48 |  |
| 32 | Q5ZU36 | Lpg1972 | 126 | 20 | 106 |  |
| 33 | Q5ZYX7 | Lpg0234 (SidE) | 107 | 0 | 107 |  |
| 34 | Q5ZTL3 | Lpg2148 (MvcA) | 84 | 34 | 50 |  |
| 35 | Q5ZX35 | Lpg0898 | 65 | 0 | 65 |  |
| 36 | Q5ZVC0 | Lpg1520 | 64 | 18 | 46 |  |
| 37 | Q5ZSL0 | Lpg2507 | 50 | 4 | 46 |  |
| 38 | Q5ZTK5 | Lpg2156 (SdeB) | 22 | 5 | 17 |  |

Note: Effectors known to be involved in ubiquitin signaling were highlighted in red.
