# Supplementary material for: Regulation of host immunity by a novel Legionella pneumophila E3 ubiquitin ligase
Source: PLoS Pathog. 2025 Sep 15;21(9):e1013522. doi: 10.1371/journal.ppat.1013522 (PMC12445743; doi:10.1371/journal.ppat.1013522)
Supplement: S2 Table — (DOCX) [file ppat.1013522.s009.docx]

**S2 Table C****andidate** **targets of Lug14 identified by UBAITs**

| Rank | Accession | Description | Total spectral counts | Unique spectral counts | | |
| --- | --- | --- | --- | --- | --- | --- |
|  |  |  |  | Lug14 | RavN | |
| 1 | P52732 | Kinesin-like protein KIF11 OS=Homo sapiens OX=9606 GN=KIF11 PE=1 SV=2 | 2408 | 1170 | 1238 |  |
| 2 | P35580 | Myosin-10 OS=Homo sapiens OX=9606 GN=MYH10 PE=1 SV=3 | 695 | 505 | 190 |  |
| 3 | P35579 | Myosin-9 OS=Homo sapiens OX=9606 GN=MYH9 PE=1 SV=4 | 409 | 295 | 114 |  |
| 4 | P0CG47 | Polyubiquitin-B OS=Homo sapiens OX=9606 GN=UBB PE=1 SV=1 | 272 | 132 | 140 |  |
| 5 | Q00839 | Heterogeneous nuclear ribonucleoprotein U OS=Homo sapiens OX=9606 GN=HNRNPU PE=1 SV=6 | 246 | 127 | 119 |  |
| 6 | P04264 | Keratin, type II cytoskeletal 1 OS=Homo sapiens OX=9606 GN=KRT1 PE=1 SV=6 | 218 | 107 | 111 |  |
| 7 | P07437 | Tubulin beta chain OS=Homo sapiens OX=9606 GN=TUBB PE=1 SV=2 | 159 | 72 | 87 |  |
| 8 | Q6P3W7 | SCY1-like protein 2 OS=Homo sapiens OX=9606 GN=SCYL2 PE=1 SV=1 | 158 | 76 | 82 |  |
| 9 | P68371 | Tubulin beta-4B chain OS=Homo sapiens OX=9606 GN=TUBB4B PE=1 SV=1 | 147 | 68 | 79 |  |
| 10 | P13645 | Keratin, type I cytoskeletal 10 OS=Homo sapiens OX=9606 GN=KRT10 PE=1 SV=6 | 138 | 65 | 73 |  |
| 11 | P35527 | Keratin, type I cytoskeletal 9 OS=Homo sapiens OX=9606 GN=KRT9 PE=1 SV=3 | 138 | 59 | 79 |  |
| 12 | Q9NYF8 | Bcl-2-associated transcription factor 1 OS=Homo sapiens OX=9606 GN=BCLAF1 PE=1 SV=2 | 126 | 44 | 82 |  |
| 13 | P60709 | Actin, cytoplasmic 1 OS=Homo sapiens OX=9606 GN=ACTB PE=1 SV=1 | 125 | 87 | 38 |  |
| 14 | O14744 | Protein arginine N-methyltransferase 5 OS=Homo sapiens OX=9606 GN=PRMT5 PE=1 SV=4 | 123 | 50 | 73 |  |
| 15 | Q7Z406 | Myosin-14 OS=Homo sapiens OX=9606 GN=MYH14 PE=1 SV=2 | 117 | 99 | 18 |  |
| 16 | Q9Y2W1 | Thyroid hormone receptor-associated protein 3 OS=Homo sapiens OX=9606 GN=THRAP3 PE=1 SV=2 | 116 | 41 | 75 |  |
| 17 | P35908 | Keratin, type II cytoskeletal 2 epidermal OS=Homo sapiens OX=9606 GN=KRT2 PE=1 SV=2 | 114 | 58 | 56 |  |
| 18 | Q13885 | Tubulin beta-2A chain OS=Homo sapiens OX=9606 GN=TUBB2A PE=1 SV=1 | 112 | 52 | 60 |  |
| 19 | Q08211 | ATP-dependent RNA helicase A OS=Homo sapiens OX=9606 GN=DHX9 PE=1 SV=4 | 107 | 47 | 60 |  |
| 20 | P19338 | Nucleolin OS=Homo sapiens OX=9606 GN=NCL PE=1 SV=3 | 105 | 59 | 46 |  |
| 21 | Q9BRS2 | Serine/threonine-protein kinase RIO1 OS=Homo sapiens OX=9606 GN=RIOK1 PE=1 SV=2 | 102 | 38 | 64 |  |
| 22 | Q9NR30 | Nucleolar RNA helicase 2 OS=Homo sapiens OX=9606 GN=DDX21 PE=1 SV=5 | 100 | 51 | 49 |  |
| 23 | Q93034 | Cullin-5 OS=Homo sapiens OX=9606 GN=CUL5 PE=1 SV=4 | 98 | 97 | 1 |  |
| 24 | P23246 | Splicing factor, proline- and glutamine-rich OS=Homo sapiens OX=9606 GN=SFPQ PE=1 SV=2 | 97 | 36 | 61 |  |
| 25 | Q9UM54 | Unconventional myosin-VI OS=Homo sapiens OX=9606 GN=MYO6 PE=1 SV=4 | 97 | 96 | 1 |  |
| 26 | O94832 | Unconventional myosin-Id OS=Homo sapiens OX=9606 GN=MYO1D PE=1 SV=2 | 90 | 88 | 2 |  |
| 27 | O95376 | E3 ubiquitin-protein ligase ARIH2 OS=Homo sapiens OX=9606 GN=ARIH2 PE=1 SV=1 | 87 | 73 | 14 |  |
| 28 | Q13509 | Tubulin beta-3 chain OS=Homo sapiens OX=9606 GN=TUBB3 PE=1 SV=2 | 86 | 39 | 47 |  |
| 29 | P68363 | Tubulin alpha-1B chain OS=Homo sapiens OX=9606 GN=TUBA1B PE=1 SV=1 | 84 | 39 | 45 |  |
| 30 | P49327 | Fatty acid synthase OS=Homo sapiens OX=9606 GN=FASN PE=1 SV=3 | 84 | 45 | 39 |  |
| 31 | P11388 | DNA topoisomerase 2-alpha OS=Homo sapiens OX=9606 GN=TOP2A PE=1 SV=3 | 84 | 42 | 42 |  |
| 32 | P21333 | Filamin-A OS=Homo sapiens OX=9606 GN=FLNA PE=1 SV=4 | 84 | 67 | 17 |  |
| 33 | O00159 | Unconventional myosin-Ic OS=Homo sapiens OX=9606 GN=MYO1C PE=1 SV=4 | 84 | 83 | 1 |  |
| 34 | P68133 | Actin, alpha skeletal muscle OS=Homo sapiens OX=9606 GN=ACTA1 PE=1 SV=1 | 83 | 60 | 23 |  |
| 35 | P02533 | Keratin, type I cytoskeletal 14 OS=Homo sapiens OX=9606 GN=KRT14 PE=1 SV=4 | 81 | 45 | 36 |  |
| 36 | P07814 | Bifunctional glutamate/proline--tRNA ligase OS=Homo sapiens OX=9606 GN=EPRS1 PE=1 SV=5 | 79 | 37 | 42 |  |
| 37 | P08779 | Keratin, type I cytoskeletal 16 OS=Homo sapiens OX=9606 GN=KRT16 PE=1 SV=4 | 74 | 41 | 33 |  |
| 38 | P35749 | Myosin-11 OS=Homo sapiens OX=9606 GN=MYH11 PE=1 SV=3 | 72 | 45 | 27 |  |
| 39 | Q13200 | 26S proteasome non-ATPase regulatory subunit 2 OS=Homo sapiens OX=9606 GN=PSMD2 PE=1 SV=3 | 71 | 41 | 30 |  |
| 40 | P04259 | Keratin, type II cytoskeletal 6B OS=Homo sapiens OX=9606 GN=KRT6B PE=1 SV=5 | 71 | 38 | 33 |  |
| 41 | P11387 | DNA topoisomerase 1 OS=Homo sapiens OX=9606 GN=TOP1 PE=1 SV=2 | 71 | 37 | 34 |  |
| 42 | O75533 | Splicing factor 3B subunit 1 OS=Homo sapiens OX=9606 GN=SF3B1 PE=1 SV=3 | 70 | 37 | 33 |  |
| 43 | P02538 | Keratin, type II cytoskeletal 6A OS=Homo sapiens OX=9606 GN=KRT6A PE=1 SV=3 | 63 | 35 | 28 |  |
| 44 | P09874 | Poly [ADP-ribose] polymerase 1 OS=Homo sapiens OX=9606 GN=PARP1 PE=1 SV=4 | 62 | 50 | 12 |  |
| 45 | P13647 | Keratin, type II cytoskeletal 5 OS=Homo sapiens OX=9606 GN=KRT5 PE=1 SV=3 | 61 | 33 | 28 |  |
| 46 | Q02880 | DNA topoisomerase 2-beta OS=Homo sapiens OX=9606 GN=TOP2B PE=1 SV=3 | 61 | 28 | 33 |  |
| 47 | Q7Z6Z7 | E3 ubiquitin-protein ligase HUWE1 OS=Homo sapiens OX=9606 GN=HUWE1 PE=1 SV=3 | 59 | 43 | 16 |  |
| 48 | P08238 | Heat shock protein HSP 90-beta OS=Homo sapiens OX=9606 GN=HSP90AB1 PE=1 SV=4 | 55 | 28 | 27 |  |
| 49 | P05141 | ADP/ATP translocase 2 OS=Homo sapiens OX=9606 GN=SLC25A5 PE=1 SV=7 | 54 | 22 | 32 |  |
| 50 | A6NHR9 | Structural maintenance of chromosomes flexible hinge domain-containing protein 1 OS=Homo sapiens OX=9606 GN=SMCHD1 PE=1 SV=2 | 34 | 1 | 33 |  |

Note: Proteins significantly more aboundant in Lug14 samples were highlighted in red.
