# Supplementary material for: Regulation of host immunity by a novel Legionella pneumophila E3 ubiquitin ligase
Source: PLoS Pathog. 2025 Sep 15;21(9):e1013522. doi: 10.1371/journal.ppat.1013522 (PMC12445743; doi:10.1371/journal.ppat.1013522)
Supplement: S3 Table — (DOCX) [file ppat.1013522.s010.docx]

**S3 Table Bacterial strains, plasmids and primers used in this study**

| Bacterial Strains | Source | Identifier |
| --- | --- | --- |
| LP02*∆lug14* | This study | N/A |
| LP02*∆lug14*(pZL507) | This study | N/A |
| LP02*∆lug14*(pLug14) | This study | N/A |
| *E.coli* BL21(DE3) | NEB | CAT#C2527I |
| *E.coli* XL1-Blue | Agilent | CAT#200249 |

| Plasmids | Source | Identifier |
| --- | --- | --- |
| pZL507 | [82] | N/A |
| pZL507::*lug14* | This study | N/A |
| pET28a | Novagen | CAT#69864 |
| pET28a::*ub* | [34] | N/A |
| pET28a::*ub-6K* | This study | N/A |
| pET28a::*ub-11K* | This study | N/A |
| pET28a::*ub-27K* | This study | N/A |
| pET28a::*ub-29K* | This study | N/A |
| pET28a::*ub-33K* | This study | N/A |
| pET28a::*ub-48K* | This study | N/A |
| pET28a::*ub-63K* | This study | N/A |
| pET28a::flag-*ub_GG/AA_* | [36] | N/A |
| pET28a::*ubcH5c* | This study | N/A |
| pET28a::*ubcH7* | This study | N/A |
| pET28a::*lug14* | This study | N/A |
| pET28a::flag-*lug14* | This study | N/A |
| pET28a::*lug14_C58A_* | This study | N/A |
| pET28a::*lug14_C247A_* | This study | N/A |
| pET28a::*lug14_C262A_* | This study | N/A |
| pET28a::*lug14_C340A_* | This study | N/A |
| pET28a::*lug14_E279A_* | This study | N/A |
| pET28a::*lug14_N366A_* | This study | N/A |
| pET28a::*lug14_N282A_* | This study | N/A |
| pET28a::*lug14_K286A_* | This study | N/A |
| pET28a::*lug14_E362A_* | This study | N/A |
| pET28a::*lug14_Q241A_* | This study | N/A |
| pET28a::*lug14_R244A_* | This study | N/A |
| pET28a::*lug14_D276A_* | This study | N/A |
| pET28a::*lug14_Y368A_* | This study | N/A |
| pET28a::*lug14_K34R_* | This study | N/A |
| pET28a::*lug14_K65R_* | This study | N/A |
| pET28a::*lug14_K286R_* | This study | N/A |
| pET28a::*SdeA_1-200_* | This study | N/A |
| pGEX6p-1 | Cytiva | CAT#28-9546-48 |
| pGEX6p-1::*lug14* | This study | N/A |
| pGEX6p-1::*lpg1751* | This study | N/A |
| pGEX6p-1::*lpg1851* | This study | N/A |
| pGEX6p-1::*lpg0634* | This study | N/A |
| peGFPC1 | Clontech | N/A |
| GFP::*lug14* | This study | N/A |
| pCDH | Addgene | CAT#72265 |
| psPAX2 | Addgene | CAT#12260 |
| pMD2.G | Addgene | CAT#12259 |
| pCDH::gfp-*lug14-3flag-ub* | This study | N/A |
| Flag::ARIH2 | This study | N/A |
| Flag::ARIH2*_K295R_* | This study | N/A |
| Flag::ARIH2*_K306R_* | This study | N/A |
| Flag::ARIH2*_K295RK306R_* | This study | N/A |
| Flag::NLRP3 | This study | N/A |
| HA::Ub | This study | N/A |

| Primers | Sequence (Restriction enzyme sites are underlined) | Note |
| --- | --- | --- |
| pSL1001 | ctgagatctatgcgaaataataaaatg | *lug14* 5F *Bgl*II |
| pSL1002 | ctggtcgacttatagtttggccgaggt | *lug14* 3R *Sal*I-1 |
| pSL1003 | ctggtcgactagtttggccgaggttat | *lug14* 3R *Sal*I-2 |
| pSL1004 | ctggtcgactttggtatcacatatttc | *lug14*up*Sal*I knockout |
| pSL1005 | aatgatccaagttccatgtcatgaaaaaaacttggaatct | *lug14*upknockout |
| pSL1006 | agattccaagtttttttcatgacatggaacttggatcatt | *lug14*downknockout |
| pSL1007 | ctgggatccgtttggggttccaggtac | *lug14*down*Bam*HI knockout |
| pSL1008 | ctgggatccatggcgctgaaacggatt | *ubcH5c* 5F *Bam*HI |
| pSL1009 | ctggtcgactcacatggcatacttctg | *ubcH5c* 3R *Sal*I |
| pSL1010 | ccccgtaagggttttcacgaaaatctg | *ub-6K*-1 |
| pSL1011 | cagattttcgtgaaaacccttacgggg | *ub-6K*-2 |
| pSL1012 | cgagggtgatggtcttccccgtaagggtt | *ub-11K*-1 |
| pSL1013 | aacccttacggggaagaccatcaccctcg | *ub-11K*-2 |
| pSL1014 | tggatcctggcctttacattttctatcgtatccgag | *ub-27K*-1 |
| pSL1015 | ctcggatacgatagaaaatgtaaaggccaggatcca | *ub-27K*-2 |
| pSL1016 | cttccctatcctggatcttggcccttacattttct | *ub-29K*-1 |
| pSL1017 | agaaaatgtaagggccaagatccaggatagggaag | *ub-29K*-2 |
| pSL1018 | gaggaattccttccttatcctggatcctggcc | *ub-33K*-1 |
| pSL1019 | ggccaggatccaggataaggaaggaattcctc | *ub-33K*-2 |
| pSL1020 | agagactgatctttgctggcaagcagctggaaga | *ub-48K*-1 |
| pSL1021 | tcttccagctgcttgccagcaaagatcagtctct | *ub-48K*-2 |
| pSL1022 | tgaagagtagactccttttgaatattgtagtcagacaaagtacgt | *ub-63K*-1 |
| pSL1023 | acgtactttgtctgactacaatattcaaaaggagtctactcttca | *ub-63K*-2 |
| pSL1024 | gggactgtgactgcgaatggcggatattaggaggtgatat | *lug14_C58A_*-1 |
| pSL1025 | atatcacctcctaatatccgccattcgcagtcacagtccc | *lug14_C58A_*-2 |
| pSL1026 | ggtgccataactggaggcagccagtcggaatgct | *lug14_C247A_*-1 |
| pSL1027 | agcattccgactggctgcctccagttatggcacc | *lug14_C247A_*-2 |
| pSL1028 | caacagaattcatcatgaattgaatggcttccattgcttcttcgtagtaaatag | *lug14_C262A_*-1 |
| pSL1029 | ctatttactacgaagaagcaatggaagccattcaattcatgatgaattctgttg | *lug14_C262A_*-2 |
| pSL1030 | ttttcgcttctttctgagcaaaatcacgaaatgaaatgaacctcttatcctg | *lug14_C340A_*-1 |
| pSL1031 | caggataagaggttcatttcatttcgtgattttgctcagaaagaagcgaaaa | *lug14_C340A_*-2 |
| pSL1032 | tttttgcagggaattttttcctgcaactaaatccgtttcactcaac | *lug14_E279A_*-1 |
| pSL1033 | gttgagtgaaacggatttagttgcaggaaaaaattccctgcaaaaa | *lug14_E279A_*-2 |
| pSL1034 | tagtaatctgagaaataaccagcacctacgttttcttgcttgatgcgtaaaa | *lug14_N366A_*-1 |
| pSL1035 | ttttacgcatcaagcaagaaaacgtaggtgctggttatttctcagattacta | *lug14_N366A_*-2 |
| pSL1037 | cttgctttttgcagggaagcttttccttcaactaaatccgtttcactcaac | *lug14_N282A_*-1 |
| pSL1038 | gttgagtgaaacggatttagttgaaggaaaagcttccctgcaaaaagcaag | *lug14_N282A_*-2 |
| pSL1039 | gtcccggacttgctgcttgcagggaattttttccttcaactaa | *lug14_K286A_*-1 |
| pSL1040 | ttagttgaaggaaaaaattccctgcaagcagcaagtccgggac | *lug14_K286A_*-2 |
| pSL1041 | gaaataaccattacctacgtttgcttgcttgatgcgtaaaagagc | *lug14_E362A_*-1 |
| pSL1042 | gctcttttacgcatcaagcaagcaaacgtaggtaatggttatttc | *lug14_E362A_*-2 |
| pSL1043 | ccagtcggaatgctgctacagtcggttgagctccc | *lug14_Q241A_*-1 |
| pSL1044 | gggagctcaaccgactgtagcagcattccgactgg | *lug14_Q241A_*-2 |
| pSL1045 | aactggagcaagccagtgcgaatgcttgtacagtcg | *lug14_R244A_*-1 |
| pSL1046 | cgactgtacaagcattcgcactggcttgctccagtt | *lug14_R244A_*-2 |
| pSL1047 | ggaattttttccttcaactaaagccgtttcactcaactcaacaga | *lug14_D276A_*-1 |
| pSL1048 | tctgttgagttgagtgaaacggctttagttgaaggaaaaaattcc | *lug14_D276A_*-2 |
| pSL1049 | tgtgcggtagtaatctgagaaagcaccattacctacgttttcttgc | *lug14_Y368A_*-1 |
| pSL1050 | gcaagaaaacgtaggtaatggtgctttctcagattactaccgcaca | *lug14_Y368A_*-2 |
| pSL1051 | ctaacaattcgctatctctaatgattaacgggtcgtctaccaata | *lug14_K34R_*-1 |
| pSL1052 | tattggtagacgacccgttaatcattagagatagcgaattgttag | *lug14_K34R_*-2 |
| pSL1053 | ccataattctttgatttaaatatctgggactgtgactgcgaatgca | *lug14_K65R_*-1 |
| pSL1054 | tgcattcgcagtcacagtcccagatatttaaatcaaagaattatgg | *lug14_K65R_*-2 |
| pSL1055 | gtcccggacttgctctttgcagggaattttttccttc | *lug14_K286R_*-1 |
| pSL1056 | gaaggaaaaaattccctgcaaagagcaagtccgggac | *lug14_K286R_*-2 |
| pSL1057 | ctgggatccatgacttttactccgccc | *lpg1751* 5F *Bam*HI |
| pSL1058 | ctggtcgactcaaacaaataaagacgt | *lpg1751* 3R *Sal*I |
| pSL1059 | ctgggatccatgtcatttgaattggtc | *lpg1851* 5F *Bam*HI |
| pSL1060 | ctggtcgacttaaaggcgatgatttgc | *lpg1851* 3R *Sal*I |
| pSL1061 | ctgggatccatgttggagactaacatgacg | *Lpg0634* 5F *Bam*HI |
| pSL1062 | ctggtcgacttatacggtcggtttgct | *Lpg0634* 3R *Sal*I |
| pSL1063 | ctgggatccatcagtttgggagaagcc | *SdeA_1-200_* 5F *Bam*HI |
| pSL1064 | ctggtcgactttttcagtgttttctct | *SdeA_1-200_* 3R *Sal*I |
| pSL1065 | ctgggatccatgtcagtggacatgaat | *Arih2* 5F *Bam*HI |
| pSL1066 | ctggtcgacttaggtgtcatggaaatc | *Arih2* 3R *Sal*I |
| pSL1067 | gcacttgggacagtctctagtgtgagcactaatgt | *Arih2_K295R_* -1 |
| pSL1068 | acattagtgctcacactagagactgtcccaagtgc | *Arih2_K295R_* -2 |
| pSL1069 | gattgcagcctccattcctctcaatgcagatgttg | *Arih2_K306R_* -1 |
| pSL1070 | caacatctgcattgagaggaatggaggctgcaatc | *Arih2_K306R_* -2 |
| pSL1071 | ctgggatccatgaagatggcaagcacc | *Nlrp3* 5F *Bam*HI |
| pSL1072 | ctggtcgacctaccaagaaggctcaaa | *Nlrp3* 3R *Sal*I |
