# Supplementary material for: Regulation of host immunity by a novel Legionella pneumophila E3 ubiquitin ligase
Source: PLoS Pathog. 2025 Sep 15;21(9):e1013522. doi: 10.1371/journal.ppat.1013522 (PMC12445743; doi:10.1371/journal.ppat.1013522)
Supplement: Attachment — Submitted filename: Liu et al response to review (PPATHOGENS-D-25-00238).docx [file ppat.1013522.s012.docx]

We wish to express our gratitude to the three expert reviewers for their constructive comments and suggestions, which have greatly improved the quality of our manuscript. We have revised our manuscript in accordance with their comments and our point-to-point response to these comments are as follows. Please note that revised texts were underlined.

**Major Issues:**

Reviewer #2:

(I) Lug14 capacity to ubiquitinate ARIH2 in a classical in vitro ubiquitination assay (Figs 5C, D and E) where all the components are in excess, which normally results in a robust substrate modification, is not impressive. Based on the data I might argue that monoubiquitinated ARIH2 is the most likely product produced by Lug14 in those reactions. Based on the data in Figs 5C, D and E, I would estimate that < 5% of ARIH2 is ubiquitinated. This is a problem because unlike in those assays, Lug14 under physiological conditions when translocated in the host cytosol by the bacterium is likely present at a very low amounts (as most Legionella effectors). The concern is that ARIH2 might not be a physiologically relevant target for Lug14. The paucity of data from NLPR3 and ARIH2 ubiquitination or functionality assay under infection conditions is not reassuring.
    Response: We agreed with the comment that Lug14-mediated ubiquitination of ARIH2 is not as robust as other bacterial E3s and their substrates (e.g. SidC of *L. pneumophila*). Yet, this “weak” activity of Lug14 toward ARIH2 may reflect the delicate balance the bacterium needs to strike during its attack proteins of multiple roles. As correctly pointed out by the reviewer (point II below), ARIH2 has multiple substrates that are involved in diverse cellular processes, complete inhibition of its activity by Lug14 may adversely affect the biogenesis and maintenance of the LCV by *L. pneumophila*.

Our conclusion that ARIH2 is a substrate of Lug14 is supported by the following lines of results:

A. ARIH2 is the only candidate from our screen using the ubiquitin-activated interaction traps (UBAITs) method that can be repeatably ubiquitinated by Lug14 in biochemical and cellular assays.
B. We have identified the sites on ARIH2 that are modified by Lug14.
C. We showed that ubiquitination by Lug14 affects its enzymatic activity as an E3 ligase.

(II) Data in Fig 6A shows that ARIH2 ubiquitinates NLRP3 despite Lug14 addition (high molecular smear above NLRP3). Although, the data is very difficult to interpret because the authors decided to append a Flag tag to both ARIH2 as well as NLRP3 and did not include sufficient experimental control conditions to determine what the single band they designate as Ub-NLRP3 actually represents. A good control in this experiment would be to include the ARIH2 K295R K306R mutant protein, which could not be ubiquitinated by Lug14. I should point out that there are ARIH2 substrates other than NLRP3 in the literature that the authors have not explored (PMIDs 22037423, 23213454, 24076655, 23179078), including Cullin-based UBLs and ikBb. Thus, it is possible that they are missing the correct substrate (especially because of lack of NLRP3 functional data – see point III)
Response: Thank you for the constructive suggestion. We have redone the experiment by adding samples with the ARIH2_K295RK306R_ mutant protein as an additional control (**Fig 6A**). Ubiquitinated NLRP3, ARIH2 and Lug14 were detected by immunoblotting with anti- NLRP3, anti-Flag and anti-GST antibodies, respectively. Our results showed that although it could not be ubiquitinated by Lug14, the ARIH2_K295RK306R_ mutant still underwent self-ubiquitination, but at a level lower than that of wild-type protein. More importantly, we observed robust ARIH2 ubiquitination in samples receiving Lug14, which was accompanied by a decrease in NLRP3 ubiquitination.

We agree that like many E3 ligases from both pathogens like *L. pneumophila* and eukaryotic cells, ARIH2 has multiple substrates. Clearly, inhibition of ARIH2 activity by Lug14 will impact the function of these substrates, thus affecting intracellular replication of *L. pneumophila*. Our current manuscript is the initial description of the identification, its catalysis and potential substrates of a novel E3 ligase from this bacterial pathogen. The full spectrum of its substrates and other aspects of its activity are of great interest and our groups are actively pursuing these lines of research. It is very common that the impact of an E3 ligase expands after active investigation over many years. For example, after the initial identification of SidC as an E3 ligase in 2014 [1], more than 10 following up papers (e.g. [2-4]) have been published to describe its mechanism of action and targets.

(III) The data demonstrating Lug14 translocated by the bacteria under infection conditions ubiquitinating ARIH2 and enhancing NLRP3 activity is lacking (Fig. 6B-C notwithstanding). The functional data in Fig 6B-C (presumably showing consequences for the NLRP3-inflammasome) are not rigorous. There are a large number of well-established molecular tools for assessment of NLRP3 activity thus a detailed analysis showing the consequences of Lug14 translocation in macrophages during infection specifically on NLRP3 functionality is needed in this work. This is critical point because depending on the situation multiple distinct inflammasomes can be triggered by Legionella in macrophages.

Response: We have seriously considered this valuable suggestion. To confirm that Lug14 translocated by the bacterium under infection conditions ubiquitinates ARIH2 and affects NLRP3 activity, we have bred to obtain an NLRC4^−/−^/NLRP3^−/−^ double-knockout mouse line and used BMDMs from these mice to examine the role of NLRP3 in *L. pneumophila-*induced secretion of mature CASP-1 (**Fig 6B-E**). We observed that BMDMs from NLRC4^−/−^ mice challenged with strain Lp02∆lug14(pLug14) led to more CASP-1 processing compared with wild-type strain (**Fig 6B and 6D**). However, there was no significant difference in secretion of mature CASP-1 between *Lug14* gene knockout and complementation strains in BMDMs from NLRC4^−/−^/NLRP3^−/−^ mice (**Fig 6C and 6E**). These results further suggest that ubiquitination of ARIH2 by Lug14 may create a condition more conductive for the activation of the NLRP3 inflammasome.

We also observed that knockout of NLRP3 did not allow more robust intracellular growth of *L. pneumophila* (**S7B and S7D Fig**), suggesting that this protein is not involved in defense against this bacterium under our experimental conditions (without priming the BMDMs with agents such as LPS). As NLRP3 is known to play roles beyond its canonical function as an inflammasome [5], we proposed that Lug14 functions to fine tune its activity to maintain cellular homeostasis required for intracellular bacterial growth. We have added a brief discussion to explain such possibility (page 23).

Reviewer #3:

Mechanistic details of Lug14's E3 ligase activity: The authors conclude that Lug14 does not require a cysteine residue for its ligase activity, suggesting a non-canonical mechanism. However, the precise catalytic mechanism remains unclear. The authors identified E362 and D276 as important residues, but additional structural or biochemical evidence would strengthen the proposed non-canonical mechanism.

Response: We agree with the point that additional structural or biochemical results are needed to understand the precise catalytic mechanism of Lug14 as an E3 ligase. Our efforts to obtain crystals of recombinant Lug14 for structural determination has not yet been successful. We have toned down the statement about the novelty of its catalytic mechanism by saying that its catalysis does not require a cysteine residue. Structural basis of its catalysis mechanism is important and interesting but it is beyond the scope of the current manuscript.

**Minor Issues:**

Reviewer #1:

(1) In Figure 3D & F, it will be better to test the mutational effects of the E362 and D276 residues on the interaction between Lug14 and Ub.
Response: We agree that it is nice to have this piece of information. We have attempted to detect the interaction between Ub and Lug14 or Lug14_E362AD276A_ by surface plasmon resonance (Biacore) but the affinity between the wild-type protein and Ub is not detectable. This result is not surprising because rarely the interaction between an E3 ligase and Ub is detectable [6].

(2) In Figure 3G, does the K11R mutation in Ub reduce the Lug14's auto-ubiquitination level, relative to wild-type Ub?
Response: We have further compared self-ubiquitination of Lug14 with ubiquitin mutants each carrying a K to R mutation by biochemical reactions. Our results showed that the K11R mutation does appear to affect self-modification of this E3 ligase (**Fig 3G, left**).
(3) In Figure 5, does Lug14 catalyze the K11-linked polyUb on ARIH2 in vitro?
Response: We have utilized Ub_K11_ in our biochemical reactions for Lug14-mediated ubiquitination of ARIH2, our results showed that ARIH2 was monoubiquitinated (**Fig 5C**).
(4) In Figure 6B, it will be better to include the infection result of the deletion strain complemented with the catalytically inactive Lug14 and its effects on NLRP3 inflammasome activation.

Response: We appreciate this very constructive suggestion. We have introduced a Lug14_E362AD276A_-expressing plasmid into strain Lp02 and examined its effects on NLRP3 inflammasome activation (**Fig 6B**). Results from infection experiments showed that strain Lp02∆*lug14*(pLug14_E362AD276A_) indeed induced less CASP-1 maturation than the strain wild-type.

Reviewer #2:

(I) The authors state that Lug14 does not conjugate Ub to a Cysteine residue for substrate transfer (a proposed novelty in enzyme functionality) because individual single Cys point mutations did not abrogate Ub acceptance. However, two of the residues C247 and C262 are sufficiently proximal that might be functionally redundant. A double mutant should be used to eliminate that possibility.
Response: We have followed the suggestion and further analyzed the E3 activity of mutants lacking all cysteine residues, the results showed that even the mutant in which all 4 Cys residues have been mutated still retained activity at levels comparable to that of wild-type protein (**Fig 3C**).
(II) From the data in Fig 3G the authors postulate that Lug14 has preference for K11 Ub chains, however given the differences in Lug14 amounts in each reaction and the lack of quantitative analysis it is hard to draw that conclusion. It is unclear if equal amounts of each Ub mutant is added in each reaction (the immunoblot for Ub is missing). I am curious, can the authors comment on why WT Ub is the least efficiently incorporated in Lug14 in those assays?

Response: Thank you for this suggestion. We have repeated the experiment. After adding all reactants into test tubes in ice, we took half of the sample in each reaction to determine the amount of Ub detected by Coomassie brilliant blue staining (**S3 Fig**) prior to switching the samples to 37°C for the reaction to proceed (**Fig 3G, right**). The amount of Ub in these reactions is equal (**S3 Fig**). We have repeated these experiments with a different batch of ubiquitin, the results indicate that wild-type Ub did not incorporate onto Lug14 less efficiently than its mutants (**Fig 3G).**
(III) The mass spec data from the proximity labelling screen should be provided as supplementary data source (including total AND unique spectral counts for each protein). In the same vein, the data in S1 table should also include unique spectral counts not just total spectral counts.
Response: We have added the mass spec data from the proximity labelling screen (**S1 Table**) and revised **S2** **Table**.

(IV) I would recommend that the intracellular growth assays in Fig 4 be presented as fold change in CFUs rather that total CFUs for easier data interpretation.
Response: We have followed the suggestion to revise **Fig 4**.
(V) Based on the OD data curve it seems to me that only log phase bacteria are sampled in S2 figure. I don’t see clear stationary phase. Also, the authors can include a control such as FlaA, which varies based on the growth phase.
Response: We have repeated the experiment and added another *L. pneumophila* effector SidC, which is known to express at higher level in the stationary phase of the culture, as a control. Whereas the amount of SidC decreased after the cells were diluted into fresh medium, the amount of Lug14 remains constant throughout the experimental duration (**S4 Fig**).
(VI) For the benefit of non-specialist readers of this manuscript, it would be informative for the authors to include a short description of the UBAIT methodology they used at around line 303.

Response: We agree with this statement and have revised the text accordingly (page 16). The text reads: “This strategy involves the construction of an E3-ubiquitin fusion that allows the C-terminus of ubiquitin to form an amide linkage to proteins potentially modified by the E3 ligase together with E1 and E2 enzymes, enabling co-purification of the E3 with candidate substrates”.

Reviewer #3:

Figure 3G: The authors conclude that Lug14 prefers K11-linked chains, but the difference between K11 and some other chain types (e.g., K6, K33) appears modest. Statistical analysis would strengthen this conclusion.

Response: We have repeated the experiment and proteins resolved by SDS-PAGE were detected by immunoblotting using Ub-specific antibody (**Fig 3G, right**). Meanwhile, we have added a panel of ubiquitin mutants, each with mutation of one lysine residue to arginine (K to R) in these reactions (**Fig 3G, left**). The results clearly show the different reaction efficiency of these Ub mutants. As for the suggestion to quantitate these bands, the fact that the signals appeared as smearing continuous bands made it difficult to determine signal intensity.

Line 247-259: The authors used AlphaFold to predict the structure of the Lug14-Ub complex. Given the importance of this prediction for identifying potential interaction sites, further details on the model quality (pLDDT scores, PAE values) would be valuable.

Response: Thank you for this suggestion. We have added the pLDDT scores and PAE values in **S6 Fig**.

**References**

1.	Hsu F, Luo X, Qiu J, Teng YB, Jin J, Smolka MB, Luo ZQ, Mao Y. The *Legionella* effector SidC defines a unique family of ubiquitin ligases important for bacterial phagosomal remodeling. Proc Natl Acad Sci U S A. 2014. **111**(29): p. 10538-43. doi: 10.1073/pnas.1402605111.

2.	Liu S, Luo J, Zhen X, Qiu J, Ouyang S, Luo ZQ. Interplay between bacterial deubiquitinase and ubiquitin E3 ligase regulates ubiquitin dynamics on *Legionella* phagosomes. Elife. 2020. **9**: e58114. doi: 10.7554/eLife.58114.

3.	Steinbach AM, Bhadkamkar VL, Jimenez-Morales D, Stevenson E, Jang GM, Krogan NJ, Swaney DL, Mukherjee S. Cross-family small GTPase ubiquitination by the intracellular pathogen *Legionella pneumophila.* bioRxiv. 2023.08.03.551750. doi: 10.1101/2023.08.03.551750.

4.	Steinbach A, Bhadkamkar V, Jimenez-Morales D, Stevenson E, Jang GM, Krogan NJ, Swaney DL, Mukherjee S. Cross-family small GTPase ubiquitination by the intracellular pathogen *Legionella pneumophila*. Mol Biol Cell. 2024. **35**(3). doi: 10.1091/mbc.E23-06-0260.

5.	Accogli T, Hibos C, Vegran F. Canonical and non-canonical functions of NLRP3. Journal of Advanced Research. 2023. **53**: p. 137-151. doi: 10.1016/j.jare.2023.01.001.

6.	Yang Q, Zhao J, Chen D, Wang Y. E3 ubiquitin ligases: styles, structures and functions. Mol Biomed. 2021. **2**(1):23. doi: 10.1186/s43556-021-00043-2.
